# Safety of Early Mobilisation in Critically Ill Patients Under Unusual High‐Risk Conditions: A Latin American Case Series

**DOI:** 10.1155/crcc/7899295

**Published:** 2026-08-14

**Authors:** Andrés Mauricio Enriquez Popayán, Ruvistay Gutierrez-Arias, Luis Alexander Peña-López, Henry Mauricio Parada-Gereda

**Affiliations:** ^1^ Department of Physiotherapy in Intensive Care, SES Hospital Universitario de Caldas, Manizales, Colombia; ^2^ Research Group GIHORO, Hospital Regional de la Orinoquia, Yopal, Colombia; ^3^ Department of Support in Comprehensive Cardiopulmonary Rehabilitation, Instituto Nacional del Torax, Santiago, Chile; ^4^ INTRehab Research Group, Instituto Nacional del Torax, Santiago, Chile; ^5^ Exercise and Rehabilitation Sciences Institute, School of Physical Therapy, Faculty of Rehabilitation Sciences, Universidad Andrés Bello, Santiago, Chile, unab.cl; ^6^ Department of Anesthesiology, Universidad del Cauca, Popayán, Colombia, unicauca.edu.co; ^7^ Intensive Care Unit, Hospital Universitario San José, Popayán, Colombia; ^8^ Research Group GRIIAS, Hospital Universitario San José, Popayán, Colombia; ^9^ Asociación Colombiana de Medicina Crítica y Cuidado Intensivo, AMCI, Bogotá, Colombia; ^10^ Intensive Care Unit Clínica Reina Sofía, Clínica Colsanitas, Clinical Nutrition and Rehabilitation Research Group, Keralty, Bogotá, Colombia

**Keywords:** antiarrhythmia agents, blood transfusion, early mobilisation, intensive care unit, parenteral nutrition, vasoconstrictor agent

## Abstract

**Background:**

Early mobilisation (EM) in the intensive care unit (ICU) is associated with a reduced incidence of intensive care unit‐acquired weakness, shorter hospital length of stay and improved functional outcomes. However, most of the available evidence derives from patient populations at low risk of safety‐related adverse events. This limits the generalisability of its benefits to more complex clinical scenarios, such as the concurrent administration of blood products, continuous parenteral nutrition, antiarrhythmic infusions or multiple haemodynamic pharmacological support. Consequently, evidence regarding the safety of EM in these contexts remains scarce. This case series is aimed at describing the safety of EM under unusual and high‐risk clinical conditions, an area that remains underexplored in the international literature.

**Case Presentation:**

We describe six critically ill patients presenting with conditions traditionally considered high risk for EM. Two patients were receiving active transfusion of blood products: a 70‐year‐old woman under invasive mechanical ventilation and a 69‐year‐old man with upper gastrointestinal bleeding and moderate anaemia; both performed active exercises during red blood cell transfusion. Two additional patients were receiving continuous enteral nutrition: a 42‐year‐old man requiring invasive mechanical ventilation and a 76‐year‐old woman; both undertook active exercises during nutritional administration. The fifth case involved a 65‐year‐old woman with atrial flutter treated with an amiodarone infusion. The sixth case was a 65‐year‐old woman with cardiomyopathy and suspected sepsis, requiring triple haemodynamic pharmacological support with noradrenaline, vasopressin and dobutamine. Exercise prescription was individualised according to intensity, type and duration, based on the dosing determined by the attending physiotherapist. All interventions were conducted under continuous monitoring. No adverse events or complications related to EM or invasive devices were observed.

**Conclusions:**

EM can be safely implemented in critically ill patients, even under conditions traditionally considered high risk, provided that strict criteria for clinical stability and continuous monitoring are applied. These findings provide relevant preliminary evidence supporting the feasibility of active rehabilitation in the ICU and open new avenues for research aimed at defining specific safety criteria for EM in high‐risk populations.

## 1. Introduction

Over recent decades, initiating early mobilisation (EM) in the intensive care unit (ICU) as soon as feasible has been associated with a reduction in intensive care unit‐acquired weakness (ICU‐AW) [1, 2], shorter hospital length of stay and improved functional outcomes [3]. However, most of this evidence derives from populations that are relatively haemodynamically stable, leaving substantial gaps in knowledge [4, 5], particularly in clinical scenarios traditionally regarded as high risk. These include patients receiving active blood product transfusions, those requiring multiple haemodynamic pharmacological supports, individuals undergoing antiarrhythmic infusions due to cardiac electrical instability and those receiving continuous enteral nutrition [6, 7]. In such contexts, EM is frequently deferred owing to concerns about adverse events, including haemodynamic decompensation, exacerbation of bleeding, impaired tissue perfusion or worsening arrhythmias. Nevertheless, absence of evidence does not equate to evidence of harm, and the risk–benefit balance remains inadequately defined.

In this article, we present a Latin American case series describing the safe implementation of EM across four unusual and highly complex clinical scenarios: (1) critically ill patients requiring blood product transfusion, (2) critically ill patients receiving continuous enteral nutrition, (3) a critically ill patient under antiarrhythmic pharmacological support and (4) a critically ill patient receiving triple haemodynamic pharmacological support. These cases provide preliminary clinical evidence in areas where the literature remains scarce or absent and aim to contribute to the development of more precise criteria to guide decision‐making in active physical rehabilitation within the ICU.

## 2. Case Presentation

All cases described were admitted to the ICU of SES Hospital Universitario de Caldas between May 2025 and March 2026. The unit admits adult patients with neurovascular, burn, cardiovascular, renal, respiratory, polytrauma and surgical conditions. It has a capacity of 20 beds. In our ICU, physiotherapists (PTs) are the professionals responsible for comprehensive rehabilitation, including physical and pulmonary rehabilitation as well as respiratory care for critically ill patients; therefore, all interventions were performed directly by the on‐duty PT. The PT‐to‐bed ratio in our unit is 1 PT per 10 patients, with 24‐h availability, 7 days a week.

### 2.1. Interventions

These interventions were performed during the daytime, between 08:00 and 17:00. All the procedures described were carried out as part of routine clinical care.

To determine when to initiate EM, the PT conducts a daily assessment of all patients admitted to the ICU. This assessment includes the evaluation of different body systems with respect to movement‐related body functions [8]. The safety criteria described below correspond to the guidelines established in our institutional protocol.

Within the neuromuscular domain, a level of consciousness greater than 12 points on the Glasgow Coma Scale or a score between −3 and +1 on the Richmond Agitation‐Sedation Scale (RASS) was required to perform active or active‐assisted interventions. Within the cardiopulmonary domain, the following parameters were verified: a heart rate (HR) between 40 and 130 beats per minute (bpm), a systolic blood pressure (BP) between 60 and 160 mmHg, a diastolic BP between 45 and 100 mmHg, a mean arterial pressure (MAP) between 45 and 110 mmHg, a respiratory rate between 10 and 30 respirations per minute (rpm), a peripheral oxygen saturation (SpO_2_) of ≥ 90%, a fraction of inspired oxygen (FiO_2_) of ≤ 60% and a positive end‐expiratory pressure (PEEP) of ≤ 10 cmH_2_O. Within the musculoskeletal domain, a score of ≥ 2 on the ICU mobility scale (IMS) and a total score of ≥ 30 on the Medical Research Council (MRC) scale were required.

Regarding laboratory variables, the protocol specified a haemoglobin concentration of ≥ 7 grams per decilitre (g/dL), a platelet count of ≥ 20,000 per microlitre (mcL), and a lactate concentration of ≤ 6 millimoles per litre (mmol/L).

In addition, any complementary investigations already available for the patient and considered relevant by the PT were reviewed to support the assessment process and clinical decision‐making. Based on the findings obtained during the assessment, the PT determines the type of intervention to be implemented, which may be passive, active‐assisted or active.

In this context, EM is initiated as soon as the patient′s clinical condition allows. However, in patients who meet the established safety criteria from the first day in the ICU, EM may be commenced immediately. The findings from this structured assessment for each patient are presented comparatively in Table 1.

**Table 1:** Physiotherapy assessment.

Cases	Domains			
	Neuromuscular	Cardiopulmonary	Musculoskeletal	Additional tests
1	Glasgow 15/15	HR: 70 bpm, BP: 121/77 mmHg, MAP: 91 mmHg, RR: 19 rpm, SpO_2_: 93%, TQT #7, ventilator settings: PS: 10 cmH_2_O, PEEP: 5 cmH_2_O, FiO_2_: 35%, Borg scale: 1/10, ETV: 359 mL, no PVA	MRC: < 45, IMS: 4/9	Hb: 7.0 g/dL, platelet count: 985,000 mcL
2	Glasgow 15/15	HR: 88 bpm, BP: 126/63 mmHg, MAP: 84 mmHg, RR: 16 rpm, SpO_2_: 92%, Borg scale: 0/10	MRC > 45, IMS 8/9	Hb: 7.5 g/dL, platelets: 359,000 mcL
3	Glasgow 14/15	HR: 81 bpm, BP: 96/72 mmHg, MAP: 80 mmHg, RR: 21 rpm, SpO_2_: 96%, TQT #8, ventilator settings: PS: 8 cmH_2_O, PEEP: 6 cmH_2_O, FiO_2_: 30%, Borg scale: 3/10, ETV: 474 mL, no PVA	MRC: < 45, IMS: 3/9	Hb: 7.8 g/dL, platelets: 500,000 mcL, glucometry: 146 mg/dL. Lactate: 1.4 mmol/L
4	Glasgow 14/15	HR: 83 bpm, BP: 105/88 mmHg, MAP: 82 mmHg, RR: 19 rpm, SpO_2_: 98%, TQT #7, FiO_2_: 24%, Borg scale: 2/10.	MRC: > 45, IMS: 8/9	Glucometry: 139 mg/dL
5	Glasgow 15/15	HR: 82 bpm, BP: 110/77 mmHg, MAP: 84 mmHg, RR: 19 rpm, SpO_2_: 91%, Borg scale: 1/10, LVEF: 60%, atrial flutter rhythm observed on the monitor.	MRC: >45, IMS: 8/9	Hb: 13 g/dL, platelets: 247,000 mcL, potassium: 4.4 mmol/L, calcium: 8.9 mmol/L
6	Glasgow 15/15	HR: 89 bpm, BP: 93/65 mmHg, MAP: 74 mmHg, RR: 20 rpm, SpO_2_: 92%, Borg scale: 0/10, capillary refill time: < 3 s, mottling score: 2/5, pitting edema: Ul 1/4, Ll 2/4, LVEF: 37%.	MRC > 45, IMS 8/9	Hb: 11 g/dL, platelets: 281,000 mcL, SvO_2_: 75%, potassium 3.7 mmol/L, lactate: 1.5 mmol/L

Abbreviations: >, greater than; <, less than; #, number; %, percentage; BP, blood pressure; bpm, beats per minute; cmH_2_O, centimetres of water; dL, deciliter; ETV, expiratory tidal volume; FiO_2_, fraction of inspired oxygen; g, grams; Hb, haemoglobin; HR, heart rate; IMS, ICU mobility scale; L, litre; Ll, lower limbs; LVEF, left ventricular ejection fraction; MAP, mean arterial pressure; mcL, microliters; mg, milligrammes; ml, millilitres; mmHg, millimeters of mercury; mmol, millimoles; MRC, Medical Research Council; PEEP, positive end‐expiratory pressure; PS, pressure support; PVA, patient‐ventilator asynchrony; rpm, respiration per minute; RR, respiratory rate; SpO_2_, peripheral oxygen saturation; SvO_2_, venous oxygen saturation; TQT, tracheostomy; Ul, upper limbs.

Exercise prescription was individualised in all cases, with adjustments in intensity, type, frequency and duration according to each patient′s physiological and functional profile. Details of the prescriptions are presented in Table 2.

**Table 2:** Early mobilisation prescription.

Exercise prescription						
Variables	Case 1	Case 2	Case 3	Case 4	Case 5	Case 6
Intensity	Borg scale: 3/10	Borg scale: 4/10	Borg scale: 4/10	Borg scale: 6/10	Borg scale: 4/10	Borg scale: 3/10
Time	10 min	15 min	10 min	30 min	20 min	15 min
Type of exercise	Aerobic	Aerobic	Aerobic	Aerobic	Aerobic	Aerobic

### 2.2. Recording of Adverse Events

All adverse events related to patient safety during EM were recorded. These were classified into three categories: (1) haemodynamic and respiratory events, defined as any episode of clinical instability that prevented the continuation of EM; (2) events related to the patient′s physical integrity, including falls or any type of trauma and (3) device‐related events, defined as the accidental removal, displacement or unintended malfunction of any device used for patient monitoring or clinical support.

This study was conducted with written informed consent obtained from the patients or their relatives, authorising the review of medical records and photographic documentation. It was also approved by the Scientific Committee of the SES Hospital Universitario de Caldas.

### 2.3. Critically Ill Patient Requiring Blood Product Transfusion

#### 2.3.1. Case #1

A 70‐year‐old female with a history of bronchiectasis, oxygen‐dependent chronic obstructive pulmonary disease, acute myocardial infarction and left breast cancer treated with chemotherapy and surgery. She presented to the emergency department with abdominal pain, haematemesis and haemoptysis.

During hospitalisation, she developed episodes of atrial fibrillation with rapid ventricular response, requiring electrical cardioversion on two occasions. Due to persistent tachyarrhythmia, amiodarone infusion and vasopressor support were initiated. Owing to neurological and respiratory deterioration, the airway was secured via orotracheal intubation, and invasive mechanical ventilation (MV) was commenced.

On ICU admission, vital signs were as follows: Heart Rate (HR) 57 beats per minute (bpm), BP 99/53 mmHg, MAP 68 mmHg, respiratory rate (RR) 16 respirations per minute (rpm), and SpO_2_ 96% on invasive MV with a FiO_2_ of 35%.

During her ICU stay, the patient showed a favourable clinical course. However, after multiple failed attempts at ventilator weaning, a percutaneous tracheostomy was performed on Day 15. Liberation from MV was complex, requiring 77 days of MV and high‐flow therapy before successful weaning was achieved. The patient commenced active‐assisted EM on Day 20 of her hospital stay.

**Figure 1:**
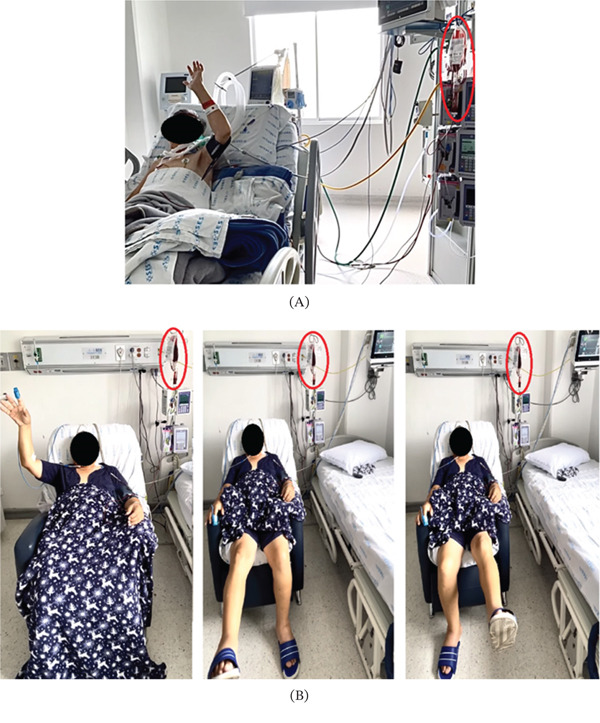
Early mobilisation during blood product transfusion. (A) Active upper‐limb exercises performed during packed red blood cell transfusion in invasive mechanical ventilation via tracheostomy. (B) Active upper‐ and lower‐limb exercises performed during packed red blood cell transfusion in a spontaneously breathing patient.

At the time of the intervention, the patient was in a semi‐Fowler′s position (35°–45°), receiving invasive MV via tracheostomy and undergoing transfusion of packed red blood cells through a peripheral venous access. There were no signs of active externalisation or evidence of gastrointestinal bleeding. In the context of clinical stability, therapeutic exercises were prescribed, including unilateral shoulder flexion activities (Figure 1A, Table 2).

No adverse events related to either the intervention or the transfusion were reported, such as catheter interruption or occlusion. Postintervention vital signs are presented in Table 3.

**Table 3:** Physiotherapy assessment following completion of the early mobilisation intervention.

Cases	Vital signs			
	Heart rate	Blood pressure	Respiratory rate	Peripheral oxygen saturation
1	HR: 96 bpm	BP: 125/65 mmHg, MAP: 84 mmHg	RR: 20 rpm	SpO_2_: 91%
2	HR: 90 bpm	BP: 119/71 mmHg, MAP: 87 mmHg	RR: 17 rpm	SpO_2_: 92%
3	HR: 80 bpm	BP: 92/69 mmHg, MAP: 76 mmHg	RR: 24 rpm	SpO_2_: 95%
4	HR: 93 bpm	BP: 115/91 mmHg, MAP: 99 mmHg	RR 26 rpm	SpO_2_: 91%
5	HR: 107 bpm	BP: 134/70 mmHg, MAP: 84 mmHg	RR: 21 rpm	SpO_2_: 93%.
6	HR: 84 bpm	BP 66/52 mmHg, MAP: 57 mmHg	RR: 19 rpm	SpO_2_: 96%.

Abbreviations: %, percentage; BP, blood pressure; bpm, beats per minute; HR, heart rate; MAP, mean arterial pressure; mmHg, millimeters of mercury; rpm, respiration per minute; RR, respiratory rate; SpO_2_, peripheral oxygen saturation.

#### 2.3.2. Case #2

A 69‐year‐old male with a history of arterial hypertension, former smoking, prior prostatectomy and suspected advanced neoplasia with nodal involvement. He was undergoing endoscopic evaluation for persistent upper gastrointestinal bleeding and presented to the emergency department following episodes of presyncope at home.

Initial assessment revealed the following vital signs: HR 111 bpm, BP 71/54 mmHg, MAP 60 mmHg, RR 18 rpm and SpO_2_ 90% on supplemental oxygen via nasal cannula FiO_2_ of 28%, without need for ventilatory support. Laboratory investigations demonstrated moderate anaemia, with haemoglobin of 8.8 g/dL, prompting transfusion of one unit of packed red blood cells and one unit of plasma.

He was transferred to the ICU for monitoring and further diagnostic work‐up, without requiring vasopressor or inotropic support. Endoscopic evaluation revealed a tumour‐like lesion extending from the cardia to the mid‐gastric antrum. The patient began an active and active‐assisted EM programme on Day 2 of her hospital stay.

Prior to the intervention, the patient was seated in a chair, receiving transfusion of packed red blood cells via a central venous catheter, with no signs of active bleeding (Table 1).

In the context of haemodynamic stability, active mobilisation exercises involving unilateral upper and lower limbs were prescribed (Figure 1B, Table 2).

No adverse events were documented following the intervention. The vital signs measured after the intervention are presented in Table 3.

### 2.4. Critically Ill patient Receiving Continuous Enteral Nutrition

#### 2.4.1. Case #3

A 42‐year‐old male with a history of arterial hypertension was admitted following high‐energy polytrauma secondary to a road traffic accident, with a diagnosis of severe traumatic brain injury. He was initially assessed at a primary care centre with a Glasgow Coma Scale score of 8/15; orotracheal intubation was not feasible, and the airway was secured using a laryngeal mask.

Upon arrival at the emergency department, vital signs were as follows: HR 128 bpm, BP 128/71 mmHg, MAP 90 mmHg, RR 22 rpm and SpO_2_ 95%. Orotracheal intubation was performed, and invasive MV was initiated. Computed tomography revealed a left frontoparietal haemorrhagic contusion and pulmonary involvement, with no abdominal injury. Neurocritical management was initiated, including deep sedation and analgesia, anticonvulsant prophylaxis and targeted strategies to optimise cerebral perfusion.

During his ICU stay, the patient initially exhibited an unfavourable course, with progressive deterioration in oxygenation, worsening radiological findings and persistent systemic inflammatory response. He required deep sedation, neuromuscular blockade and prone ventilation due to arterial partial pressure of oxygen (PaO_2_)/FiO_2_ ratio < 150 mmHg. After seven cycles of prone positioning, clinical progress remained slow and static. Owing to prolonged MV, a percutaneous tracheostomy was performed on Day 10. A total of 24 days of MV were required to achieve successful liberation from ventilatory support. The patient commenced active‐assisted EM after 15 days of hospitalisation.

Prior to the intervention, the patient was seated in a chair, receiving invasive MV via tracheostomy and continuous enteral nutrition at 46 millilitres per hour (mL/h) via a nasogastric tube (Table 1).

**Figure 2:**
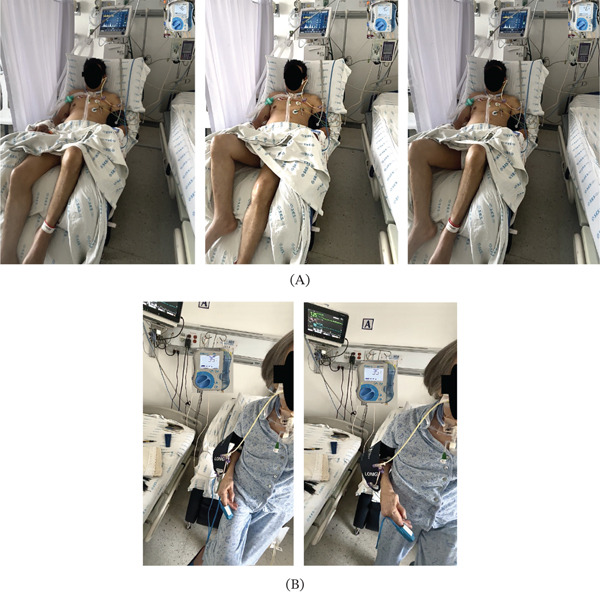
Early mobilisation during continuous enteral nutrition. (A) Lower‐limb exercises performed while receiving continuous enteral nutrition under invasive mechanical ventilation via tracheostomy. (B) Standing exercises performed while receiving continuous enteral nutrition under supplemental oxygen support via tracheostomy.

In the context of clinical stability, therapeutic exercises targeting the lower limbs were prescribed (Figure 2A, Table 2).

No adverse events were reported, including interruption of enteral nutrition or episodes of emesis during or after the intervention. The patient′s postintervention vital signs are shown in Table 3.

#### 2.4.2. Case #4

A 76‐year‐old female with a history of primary hypothyroidism, rheumatoid arthritis, osteoporosis, arterial hypertension and ischaemic heart disease presented to the emergency department with a 1‐week history of dyspnoea, weakness, asthenia, adynamia and hyporexia.

Initial evaluation revealed the following vital signs: HR 75 bpm, BP 111/71 mmHg, MAP 84 mmHg, RR 21 rpm and SpO_2_ 90%. Imaging studies demonstrated pleural effusion and pulmonary nodules suggestive of malignancy. A right‐sided thoracentesis was performed, without evidence of exudate.

The patient was evaluated by thoracic surgery and underwent video‐assisted thoracoscopic surgery, which revealed a pulmonary nodule, umbilication of the visceral pleura and mild thickening of the parietal pleura. Resection of the nodule was performed via nonanatomical segmental lobectomy, along with pleurectomy and pleurodesis, requiring right‐sided thoracostomy.

In the immediate postoperative period, she developed low cardiac output and shock, necessitating haemodynamic pharmacological support. Transthoracic echocardiography demonstrated segmental wall motion abnormalities and left ventricular dysfunction with an ejection fraction of 34%, consistent with extension of prior ischaemia. Emergency coronary angiography confirmed two‐vessel coronary artery disease, and percutaneous coronary intervention with drug‐eluting stent placement was performed.

She was admitted to the ICU in cardiogenic shock. Following clinical stabilisation, extubation was attempted; however, after failed weaning attempts, a percutaneous tracheostomy was performed on Day 9. The weaning process was prolonged, requiring a total of 34 days of MV, interspersed with high‐flow oxygen therapy, before successful liberation from ventilatory support. The active‐assisted EM intervention was initiated for the patient from Day 18 of her hospital stay.

Prior to the intervention, the patient was seated in a chair, receiving supplemental oxygen via Venturi mask with an FiO_2_ of 24%, and continuous enteral nutrition at 35 mL/h via a nasogastric tube (Table 1).

In the context of clinical stability, therapeutic exercises in the standing position were prescribed (Figure 2B, Table 2).

No adverse events related to enteral nutrition or episodes of emesis were reported. The vital signs recorded following the intervention are presented in Table 3.

### 2.5. Critically Ill Patient Receiving Antiarrhythmic Pharmacological Support

#### 2.5.1. Case #5

A 65‐year‐old female with a history of atrial septal defect closure and multiple episodes of tachycardia treated with ablation initially presented to a primary care hospital, where the electrocardiogram (ECG) documented supraventricular tachycardia. She received two doses of metoprolol and 150 milligram of intravenous amiodarone, after which she developed bradycardia to 40 bpm, prompting referral to our institution.

On admission to the ICU, vital signs were as follows: HR 98 bpm, BP 110/63 mmHg, MAP 78 mmHg, RR 18 rpm and SpO_2_ 90% on room air. Admission ECG showed sinus rhythm with frequent supraventricular extrasystoles. The electrophysiology team recommended discontinuation of propafenone and beta‐blockers, continuation of oral amiodarone and Holter monitoring, which revealed atrial flutter.

The patient commenced active EM on Day 2 of her hospital stay. At the time of assessment, the patient was seated in a chair, receiving an amiodarone infusion at 0.5 micrograms/minute. She was asymptomatic from a cardiopulmonary perspective, required no vasoactive or inotropic support and showed no evidence of active bleeding (Table 1).

In the context of clinical stability, an exercise programme was prescribed, including transitions from sitting to standing, active exercises of the upper and lower limbs and assisted marching in place (Table 2).

At the end of the intervention, the patient remained seated in a chair. The patient postintervention vital signs are shown in Table 3.

### 2.6. Critically Ill Patient Receiving Triple Haemodynamic Pharmacological Support

#### 2.6.1. Case #6

A 65‐year‐old female with a history of cerebrovascular disease, heart failure syndrome, cardiomyopathy, typical atrial flutter and a cardiac resynchronisation therapy device presented with abdominal pain and weight loss. Computed tomography of the abdomen and pelvis revealed a malignant adnexal neoplasm with features of peritoneal carcinomatosis and nodal metastases.

During hospitalisation, she underwent paracentesis for ascites. Given her cardiovascular comorbidities, cardiology and electrophysiology consultations were requested for device reprogramming prior to potential surgical intervention, and she was subsequently referred to our institution.

She was initially admitted to a general ward and later transferred to the ICU due to persistent hypotension with suspected sepsis, requiring vasopressor support with vasopressin, noradrenaline and dobutamine. At the time of clinical assessment, vital signs were as follows: HR 90 bpm, BP 61/48 mmHg, MAP: 52 mmHg, RR 18 rpm and SpO_2_ 93% on nasal cannula with an FiO_2_ of 28%.

The patient commenced active and active‐assisted EM on Day 2 of her hospital stay as part of her rehabilitation programme. Prior to the intervention, the patient was in a semi‐Fowler′s position, receiving continuous infusions of noradrenaline at 0.07 micrograms (µg)/ kilogram (kg)/ minute (min), dobutamine at 2.5 µg/kg/min, and vasopressin at 2 international units/hour (Table 1).

**Figure 3:**
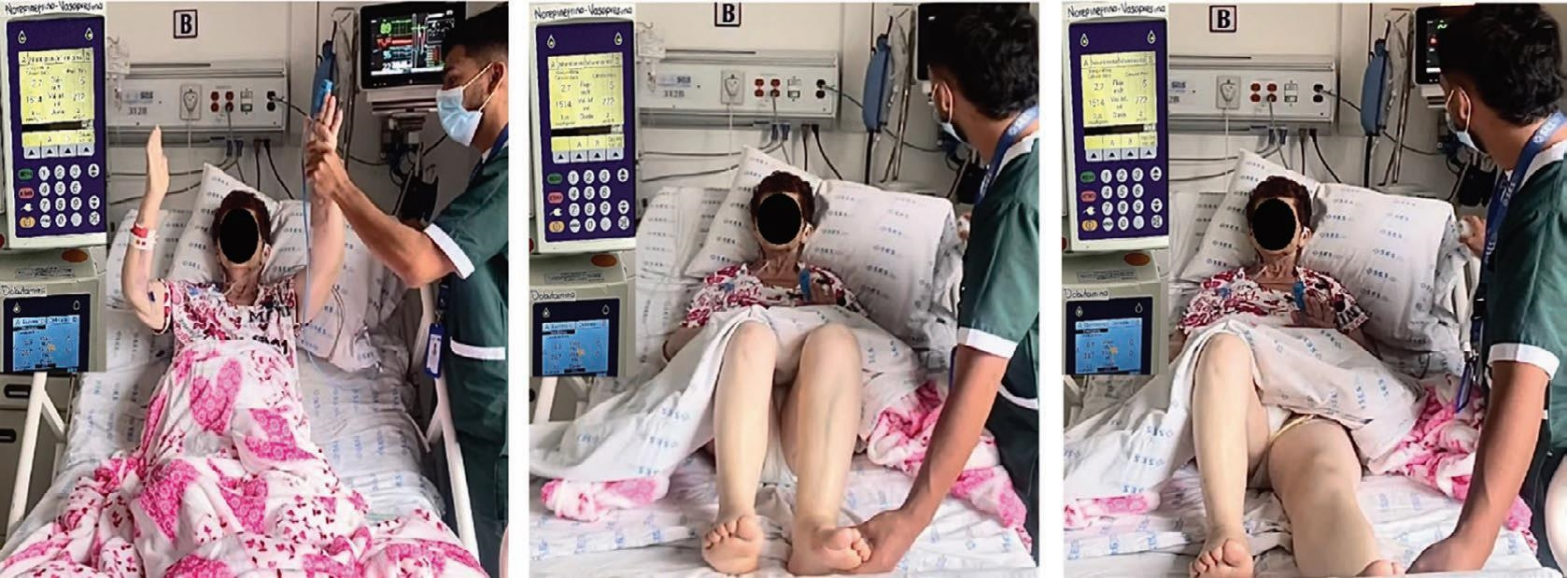
Early mobilisation during triple haemodynamic pharmacological support. Active upper‐ and lower‐limb mobilisation performed under continuous infusion of noradrenaline, vasopressin and dobutamine with continuous physiological monitoring.

She was asymptomatic from a cardiopulmonary standpoint, with no escalation in vasopressor or inotropic doses in the 30 min preceding the intervention. In the context of relative clinical stability, bilateral active mobilisation exercises of the upper and lower limbs were prescribed (Figure 3, Table 2).

No adverse events were documented following the intervention. Postintervention vital signs are presented in Table 3.

## 3. Discussion

EM in the ICU has become established as a safe and effective intervention across multiple critically ill populations [9–11]. International guidelines advocate its implementation provided that patients meet minimum clinical stability criteria [12, 13]. Nevertheless, there remain scenarios considered ‘relative contraindications’ or ‘zones of caution’, in which evidence is limited and decision‐making relies predominantly on clinical judgement.

The cases presented herein contribute to the ongoing discussion in areas where to the best of our knowledge, specific studies directly evaluating the safety of EM under certain high‐risk physiological conditions are lacking.

### 3.1. EM During Blood Product Transfusion

The cases presented in this article may represent the first report in the literature specifically addressing the safety of EM during the active administration of blood products in critically ill patients.

In the context of blood transfusions, Raasveld et al. [14] report that red blood cell transfusion is a common intervention in critically ill patients. Recent international data indicate that approximately 25% of ICU patients receive at least one transfusion during their stay, with rates varying widely depending on the setting, reaching up to 45% in some continents and as high as 80% in certain countries. Despite the frequency of this scenario in critical care, the literature appears to provide limited evidence regarding the safety of patient mobilisation during these procedures. Even the most recent international guidelines on transfusion of blood components such as packed red blood cells, plasma and platelets do not clearly address whether transfusion constitutes an absolute or relative contraindication to EM. Rather, their recommendations emphasise the need for a controlled environment, highlighting close clinical monitoring before, during and after transfusion. This includes continuous observation, particularly during the initial minutes, to ensure early detection of adverse reactions, as well as the presence of healthcare staff and frequent supervision throughout the procedure [15–17].

In clinical practise, transfusion of any blood product may be perceived as a reason to defer mobilisation interventions due to concerns about transfusion reactions, haemodynamic instability or bleeding risk. However, from a pathophysiological standpoint, if the patient is haemodynamically stable, shows no clinical signs of active bleeding and is under continuous monitoring, there may be no absolute contraindication to low‐ to moderate‐intensity mobilisation.

In our cases, both patients demonstrated haemodynamic stability, absence of bleeding externalisation and appropriate monitoring. The prescription of low‐intensity aerobic exercise (Borg 3/10), under close supervision, did not result in adverse events or clinically significant alterations, even in the presence of haemoglobin ≤ 7.5 g/dL in one case.

### 3.2. EM During Continuous Enteral Nutrition

The cases presented in this article may represent the first reported evidence specifically examining the safety of EM in patients receiving continuous enteral nutrition.

From both a historical and scientific perspective, it is striking that, despite decades of research on EM, including the characterisation of its potential adverse events, the safety of mobilisation in critically ill patients under continuous enteral nutritional support has not been explicitly evaluated. In this context, one of the closest contributions is that of Gutiérrez‐Arias et al.. [18], who report no evidence supporting the routine interruption of enteral feeding during chest physiotherapy in mechanically ventilated patients. This highlights a relevant knowledge gap, which the present cases begin to address.

In our series, no adverse events associated with EM were observed either in the mechanically ventilated patient or in the patient who achieved assisted marching in place, thereby providing preliminary support for the feasibility and safety of this intervention in the presence of continuous enteral nutrition. However, it is important to recognise that nutritional delivery may be interrupted by multiple factors, including imaging studies, gastrointestinal intolerance or abdominal distension [19]. Kasti et al. [20] note that such interruptions may delay the achievement of daily caloric targets; therefore, suspending enteral nutrition prior to EM without clear justification could inadvertently contribute to this delay.

Accordingly, a comprehensive and systematic assessment is recommended, including evaluation of emesis and tolerance to the nutritional regimen, with the aim of optimising nutritional support while ensuring the safe implementation of EM.

### 3.3. EM in Patients Receiving Antiarrhythmic Pharmacological Support

Cardiac patients are common in the ICU, and both supraventricular and ventricular arrhythmias represent frequent complications in the critical care setting [21]. Despite this, the present report may constitute the first description in the international literature specifically documenting the safety of EM during amiodarone infusion.

According to the most recent cardiac rehabilitation, guidelines addressing Phase I (which includes hospitalised patients), individuals with active arrhythmias or those receiving antiarrhythmic therapy require close monitoring during physical activity. Assessment should be individualised, taking into account haemodynamic status and cardiac rhythm. However, the available evidence does not clearly establish whether intravenous amiodarone administration constitutes an absolute contraindication to cardiac rehabilitation, underscoring the need for careful, case‐by‐case clinical judgement [22–24].

Clinical concern often centres on the potential to induce electrical instability, inappropriate increases in ventricular rate or haemodynamic deterioration. In our case, however, the patient exhibited clinical stability, preserved cardiac function, absence of cardiopulmonary symptoms and continuous monitoring. The carefully titrated intervention (Borg 4/10) resulted in expected physiological increases in HR and BP, without triggering arrhythmic events or requiring pharmacological adjustment.

### 3.4. EM in Patient Receiving Triple Haemodynamic Pharmacological Support

Critically ill patients frequently require vasoactive or inotropic pharmacological support to maintain haemodynamic stability [25]. In this context, a recent systematic review with meta‐analysis by Parada‐Gereda et al.. suggests that EM in patients receiving such agents is generally feasible and safe [26].

However, neither this manuscript nor the studies included within it precisely report the number of vasoactive or inotropic agents administered during EM; instead, data are largely limited to dosage. This represents a relevant gap in the literature, as active mobilisation in patients receiving multiple haemodynamic supports remains restricted and is often avoided due to perceived high clinical risk.

In contrast, the study by Enriquez et al. [27] provides more detailed insight. Among 24 patients who underwent active EM while receiving vasoactive or inotropic support, one was mobilised while on three simultaneous agents, without adverse events. This finding is consistent with the case presented herein, in which, despite triple pharmacological support, the patient was able to perform controlled physical exercise without adverse events or need for dose escalation. Although the intervention was performed in bed, these results suggest that, under strict criteria of clinical stability and continuous monitoring, the presence of multiple haemodynamic supports does not necessarily constitute a contraindication to active mobilisation.

In this scenario, comprehensive clinical assessment combined with an individualised exercise prescription allowed the feasibility of initiating active mobilisation to be established and its dosage to be adjusted according to the patient′s physiological tolerance. This approach may help mitigate complications associated with prolonged immobility, including ICU‐AW, functional decline and cardiovascular deconditioning.

### 3.5. Reflections

Despite the relevance of this manuscript, it represents a limited case series, which restricts the generalisability of the findings. The absence of a comparator group and the descriptive design preclude the establishment of causal relationships or the estimation of absolute risks. Furthermore, the results rely on rigorous clinical assessment and a highly monitored environment; therefore, they should be interpreted as preliminary evidence requiring confirmation through larger prospective studies. In addition, exploratory‐type reviews are needed to systematically map the available evidence in the areas explored.

Nonetheless, this manuscript directly addresses a critical gap in the scientific literature by examining the safety of EM in clinical scenarios historically regarded as high risk, including active blood product transfusion, continuous antiarrhythmic infusion, multiple haemodynamic support and continuous enteral nutrition. To the best of current knowledge, no previous reports have specifically and systematically evaluated these conditions during active mobilisation in critically ill patients, positioning this study as an original contribution within a poorly characterised clinical domain. Our findings align with the emergence of innovative and unconventional approaches to EM, such as extubation performed out of bed in a seated position on a chair [28, 29]. This evolving body of evidence, in conjunction with our results, suggests that clinical decision‐making may benefit from moving beyond strictly predefined scenarios or traditional paradigms, towards a more nuanced approach grounded in comprehensive, dynamic and individualised physiological assessment of the critically ill patient.

The findings presented challenge the traditional interpretation of these conditions as absolute contraindications to EM. On the contrary, our work suggests that, under strict criteria of clinical stability and continuous monitoring, active mobilisation interventions can be safely implemented without an increase in adverse events. These results support a shift from a restrictive approach centred on the presence of devices or therapeutic supports towards a decision‐making model grounded in comprehensive physiological assessment of the patient.

From a clinical perspective, this paradigm shift may have important implications for practise, enabling broader inclusion of patients in early rehabilitation programmes, with the potential to mitigate complications associated with prolonged immobility, such as ICU‐acquired weakness. Collectively, these findings not only expand the current boundaries of EM in critical care but also lay the groundwork for future research aimed at establishing more precise, evidence‐based criteria for its implementation in high‐risk populations.

## 4. Conclusions

EM can be safely implemented in critically ill patients, even in contexts traditionally considered high risk, provided it is grounded in a comprehensive clinical assessment, strict criteria of physiological stability and continuous monitoring. The findings of this case series provide relevant preliminary evidence supporting the feasibility and tolerability of active rehabilitation in the ICU, without an apparent increase in adverse events in underexplored scenarios. Furthermore, these results contribute to challenging restrictive approaches based solely on the presence of supports or concurrent interventions, and suggest the need to move towards more individualised decision‐making models. In this regard, new avenues for research are opened, aimed at more precisely defining safety criteria, dosing and patient selection for EM in high‐risk populations.

## Author Contributions

A.M.E.P.: conceptualization, investigation, methodology, project administration, supervision, writing—original draft and writing—review and editing. R.G‐A.: conceptualization, methodology, writing—original draft and writing—review and editing. L.A.P‐L.: conceptualization, methodology, writing—original draft and writing—review and editing. H.M.P‐G.: conceptualization, methodology, writing—original draft and writing—review and editing.

## Funding

No funding was received for this manuscript.

## Disclosure

All authors have completed the ICMJE uniform disclosure form.

## Ethics Statement

This study was conducted with written informed consent obtained from the patients or their relatives, authorising the review of medical records and photographic documentation. It was also approved by the Scientific Committee of the SES Hospital Universitario de Caldas.

## Conflicts of Interest

The authors declare no conflicts of interest.

## Data Availability

The data that support the findings of this study are available from the corresponding author upon reasonable request.

## References

[1] Khan S. A. , Moeed A. , Mari T. , Yousuf Z. , Hanson A. , Dong Y. , Cornelius P. , Anjum H. , Ratnani I. , and Surani S. , Safety and Early Mobilization in Intensive Care Unit Patients: An Updated Systematic Review and Meta-Analysis of Randomized Controlled Trials, World Journal of Critical Care Medicine. (2025) 14, no. 4, 107396, 10.5492/wjccm.v14.i4.107396, 41377548. 41377548 PMC12687039

[2] Hiser S. L. , Casey K. , Nydahl P. , Hodgson C. L. , and Needham D. M. , Intensive Care Unit Acquired Weakness and Physical Rehabilitation in the ICU, British Medical Journal. (2025) 388, e077292, 10.1136/bmj-2023-077292. 39870417

[3] Zhang L. , Hu W. , Cai Z. , Liu J. , Wu J. , Deng Y. , Yu K. , Chen X. , Zhu L. , Ma J. , and Qin Y. , Early Mobilization of Critically Ill Patients in the Intensive Care Unit: A Systematic Review and Meta-Analysis, PLoS One. (2019) 14, no. 10, e0223185, 10.1371/journal.pone.0223185, 31581205. 31581205 PMC6776357

[4] Singam A. , Mobilizing Progress: A Comprehensive Review of the Efficacy of Early Mobilization Therapy in the Intensive Care Unit, Cureus. (2024) 16, no. 4, e57595, 10.7759/cureus.57595, 38707138. 38707138 PMC11069628

[5] Zhang W. , Yu P. , Yu M. , and Mi J. , Effect of Early Goal Directed Mobilization on Clinical Outcomes in Mechanically Ventilated ICU Patients, Scientific Reports. (2025) 15, no. 1, 45399, 10.1038/s41598-025-28991-7, 41298865. 41298865 PMC12748879

[6] Malaysian Society of Intensive Care , ICU Protocol Management, 2020, Malaysian Society of Intensive Care.

[7] Society of Critical Care Medicine , Guidelines for the Management of Critically Ill Adults in The Intensive Care Unit, 2024, SCCM.

[8] World Health Organization , International Classification of Functioning, Disability and Health: ICF, 2001, World Health Organization.

[9] Nydahl P. , Sricharoenchai T. , Chandra S. , Kundt F. S. , Huang M. , Fischill M. , and Needham D. M. , Safety of Patient Mobilization and Rehabilitation in the Intensive Care Unit: Systematic Review With Meta-Analysis, Annals of the American Thoracic Society. (2017) 14, no. 5, 766–777, 10.1513/AnnalsATS.201611-843SR, 28231030. 28231030

[10] Eggmann S. , Nydahl P. , Gosselink R. , and Bissett B. , We Need to Talk About Adverse Events During Physical Rehabilitation in Critical Care Trials, EClinicalMedicine. (2024) 68, 102439, 10.1016/j.eclinm.2024.102439, 38328754. 38328754 PMC10847053

[11] Eggmann S. , Irincheeva I. , Luder G. , Verra M. L. , Moser A. , Bastiaenen C. H. G. , and Jakob S. M. , Cardiorespiratory Response to Early Rehabilitation in Critically Ill Adults: A Secondary Analysis of a randomised Controlled Trial, PLoS One. (2022) 17, no. 2, e0262779, 10.1371/journal.pone.0262779, 35113899. 35113899 PMC8812982

[12] Unoki T. , Hayashida K. , Kawai Y. , Taito S. , Ando M. , Iida Y. , Kasai F. , Kawasaki T. , Kozu R. , Kondo Y. , Saitoh M. , Sakuramoto H. , Sasaki N. , Saura R. , Nakamura K. , Ouchi A. , Okamoto S. , Okamura M. , Kuribara T. , Kuriyama A. , Matsuishi Y. , Yamamoto N. , Yoshihiro S. , Yasaka T. , Abe R. , Iitsuka T. , Inoue H. , Uchiyama Y. , Endo S. , Okura K. , Ota K. , Otsuka T. , Okada D. , Obata K. , Katayama Y. , Kaneda N. , Kitayama M. , Kina S. , Kusaba R. , Kuwabara M. , Sasanuma N. , Takahashi M. , Takayama C. , Tashiro N. , Tatsuno J. , Tamura T. , Tamoto M. , Tsuchiya A. , Tsutsumi Y. , Nagato T. , Narita C. , Nawa T. , Nonoyama T. , Hanada M. , Hirakawa K. , Makino A. , Masaki H. , Matsuki R. , Matsushima S. , Matsuda W. , Miyagishima S. , Moromizato M. , Yanagi N. , Yamauchi K. , Yamashita Y. , Yamamoto N. , Liu K. , Wakabayashi Y. , Watanabe S. , Yonekura H. , Nakanishi N. , Takahashi T. , Nishida O. , and The Committee for the Clinical Practice Guidelines of Early Mobilization and Rehabilitation in Intensive Care of the Japanese Society of Intensive Care Medicine , Japanese Clinical Practice Guidelines for Rehabilitation in Critically Ill Patients 2023 (J-ReCIP 2023), Journal of Intensive Care. (2023) 11, no. 1, 10.1186/s40560-023-00697-w, 37932849. PMC10629099 37932849

[13] Hodgson C. L. , Broadley T. , Paton M. , Higgins A. M. , Anderson S. , Brennan S. , Granger C. L. , Hammond N. E. , Magana Cruz S. , Lang J. K. , Leditschke I. A. , Orford N. R. , Parry S. M. , Price B. , Taylor P. , Udy A. A. , and Green S. E. , Australian Clinical Practice Guideline for Physical Rehabilitation and Mobilisation in Adult Intensive Care Units, Australian Critical Care. (2025) 38, no. 4, 101235, 10.1016/j.aucc.2025.101235, 40306022. 40306022

[14] Raasveld S. J. , de Bruin S. , Reuland M. C. , van den Oord C. , Schenk J. , Aubron C. , Bakker J. , Cecconi M. , Feldheiser A. , Meier J. , Müller M. C. A. , Scheeren T. W. L. , McQuilten Z. , Flint A. , Hamid T. , Piagnerelli M. , Tomić Mahečić T. , Benes J. , Russell L. , Aguirre-Bermeo H. , Triantafyllopoulou K. , Chantziara V. , Gurjar M. , Myatra S. N. , Pota V. , Elhadi M. , Gawda R. , Mourisco M. , Lance M. , Neskovic V. , Podbregar M. , Llau J. V. , Quintana-Diaz M. , Cronhjort M. , Pfortmueller C. A. , Yapici N. , Nielsen N. D. , Shah A. , de Grooth H. J. , Vlaar A. P. J. , InPUT Study Group , Higgins A. , Serpa Neto A. , Brady K. , Wood E. , Poole A. , Trapani T. , Young M. , Cooper J. , Secombe P. , Reece G. , Marella P. , Brewster D. , Rashid A. , Suwandarathne R. , Azad R. , Barrett J. , Turner E. , Poulter A. L. , Chen L. , Biradar V. , Whitehead C. , Peake S. , Tabah A. , O′Connor S. , Reade M. , Janssen G. , McAllister R. , Triplett K. , Bowen D. , Buscher H. , Santamaria J. , Parmar D. , Power P. , French C. , Mac Partlin M. , Islam M. M. , Haque I. U. , Roman A. , Haentjens L. , Ikic V. , Kvolik S. , Bojčić R. , Juričić K. , Duksa M. , Bílek L. , Satinsky I. , Zatloukal J. , Russell L. , Bestle M. H. , Meyhoff C. S. , Diaz-Medina A. M. , Llumiquinga V. , Aguirre-Bermeo H. , Gonzabay-Campos H. E. , Elbahnasawy M. , Chapalain X. , le Moal C. , Egreteau P. Y. , Launey Y. , Reizine F. , Boissier F. , Jean R. , Ehrmann S. , Lebas E. , Corno G. , Cailliez P. , Garçon P. , Carteaux G. , Kimmoun A. , Theodoulou D. , Aloizos S. , Papadakis E. , Tsakalis K. , Marinakis G. , Georgakas I. , Tripolitsioti P. , Nikolakopoulou S. , Papathanakos G. , Sklavou C. , Tsika E. , Mousafiri O. , Prekates A. , Micha G. , Lavrentieva A. , Aslanidis T. , Palaiologou A. , Bostantzoglou C. , Dikoudi E. , Karaouli S. , Pouriki S. , Vijayakumaran S. , Rathod D. , Kola V. R. , Jeswani D. , Gopalakrishna K. N. , Hartalkar A. , Mahmoodpoor A. , Abdulkhaleq M. , Ippolito M. , Cotoia A. , Covotta M. , Sanfilippo F. , Ishteiwy E. , Benzarti H. , Bouhuwaish A. E. M. , Abdalhadi A. , Buimsaedah A. , Abdulwahed E. A. , Tamoos K. , Younes E. , Alkamkhe A. A. S. , Biala M. , Hwili H. A. M. , Ben Hasan N. S. , Almiqlash B. A. , Altair M. , Otman R. , al Gharyani M. F. , Alkeelani O. M. , Bakeer H. , Omar Affat A. M. , Aween H. , Benamwor A. , Alsori M. , Abdelrahim N. , Abdelilah G. H. A. N. N. A. M. , Parke R. , Chen Y. , Mehrtens J. , Twardowski P. , Freebairn R. , Song R. , Gibson C. , Chen J. , Moore R. , Sol Cruz M. R. , Wludarczyk A. , Krzych Ł. , Szczukocka M. , Kubiak M. , Molsa M. , Wujtewicz M. , Wieczorek A. , Misiewska-Kaczur A. , Maslicki M. , Onichimowski D. , Mazur J. , Zatorski P. , Mota A. M. , Fernandes J. , Castro D. , Coelho E. , Paula A. , Guimarães T. , Adrião D. , Mark I. , Mušič E. , Mirković T. , Markota A. , Krope N. , Kmet M. , Forjan P. , Savli T. , Aguilar G. , González-Celdrán R. , Martínez-González E. , Díaz A. , Colomina M. J. , Hidalgo F. , Ferrando C. , Ferrandis R. , Ferrer C. , Cegarra V. , Gómez-Luque A. , Henman S. , Blomstrand D. , Risberg E. , Johansen N. , Rajala H. , Layous N. , Vlot E. A. , Erkamp M. , Juffermans N. , van Wonderen S. , Kuznecova-Keppel Hesselink L. , van Bochove V. , Acarel M. , Senturk E. , Karahan M. A. , Camkiran Firat A. , Yildiz Y. , Ekinci O. , Ozgultekin A. , Arikan H. , Kucukosman G. , Aydin B. G. , Yavuz M. , Oztas A. , Kavrut Ozturk N. , Kasapoglu U. S. , Miniksar H. , Tuncay E. , Indelen C. , Ogus H. , Erdivanli B. , Sahin A. S. , Yilmaz M. , Sayan E. , Yilmaz C. , Goksu S. , Basaran B. , Kutahya E. , Gok A. K. , Ozcan A. , Kara I. , Kartal S. , Saracoglu K. T. , Bilir Y. , Eyupoglu S. , Ertugrul Oruc N. , Issever K. , Patel J. , Clarke J. , Ma L. , Lawton T. , Sloan B. , Kannan S. , Innes R. , Carpenter M. , Newey L. , Alwagih H. , Acott C. , Hormis A. , Herdman J. , Akrama O. , Baumber R. , Khomenko O. , Khan A. , Hasan Z. , Raval J. S. , and Sutherland L. , Red Blood Cell Transfusion in the Intensive Care Unit, JAMA. (2023) 330, no. 19, 1852–1861, 10.1001/jama.2023.20737, 37824112. 37824112 PMC10570913

[15] National Blood Authority , Patient Blood Management Guideline for Adults With Critical Bleeding, 2024, National Blood Authority.

[16] Carson J. L. , Stanworth S. J. , Guyatt G. , Valentine S. , Dennis J. , Bakhtary S. , Cohn C. S. , Dubon A. , Grossman B. J. , Gupta G. K. , Hess A. S. , Jacobson J. L. , Kaplan L. J. , Lin Y. , Metcalf R. A. , Murphy C. H. , Pavenski K. , Prochaska M. T. , Raval J. S. , Salazar E. , Saifee N. H. , Tobian A. A. R. , So-Osman C. , Waters J. , Wood E. M. , Zantek N. D. , and Pagano M. B. , Red Blood Cell Transfusion, JAMA. (2023) 330, no. 19, 1892–1902, 10.1001/jama.2023.12914. 37824153

[17] Yataco A. C. , Soghier I. , Hébert P. C. , Alshahrani M. S. , Brostow M. E. , Despotovic J. M. , Griesdale D. E. G. , Hadid R. E. , Juffermans N. P. , Lin Y. , MacDonell J. , Morton R. P. , Ning S. , Stanworth S. J. , and Coz Yataco A. , Transfusion of Fresh Frozen Plasma and Platelets in Critically Ill Adults: Clinical Practice Guideline From the American College of Chest Physicians, Chest. (2025) 168, no. 3, 661–676, 10.1016/j.chest.2025.02.029. 40074060 PMC12489387

[18] Gutierrez-Arias R. , Salinas-Barahona F. , and Seron P. , Interruption of Enteral Tube Feeding During Chest Physiotherapy in Critically Ill Adults: A Scoping Review, Journal of Critical Care Medicine. (2026) 12, no. 1, 56–63, 10.2478/jccm-2026-0002, 41704337. 41704337 PMC12908989

[19] Lu X. , Wang X. , Yu W. , Cai J. , Wang Y. , Cao Y. , Dan L. , and Wang Q. , Current Status and Influencing Factors of Enteral Nutrition Interruption Among Critical Patients: A Systematic Review, Frontiers in Nutrition. (2025) 12, 1462131, 10.3389/fnut.2025.1462131, 40661675. 40661675 PMC12258065

[20] Kasti A. N. , Theodorakopoulou M. , Katsas K. , Synodinou K. , Nikolaki M. , Zouridaki A. , Fotiou S. , Kapetani A. , and Armaganidis A. , Factors Associated With Interruptions of Enteral Nutrition and the Impact on Macro- and Micronutrient Deficits in ICU Patients, Nutrients. (2023) 15, no. 4, 10.3390/nu15040917, 36839275. PMC9959226 36839275

[21] Yue S. , Li J. , and Zhang H. , Cardiac Arrhythmias in Critically Ill Patients: Epidemiology, Risk Factors, and Management, Critical Care Medicine. (2023) 51, no. 2, 215–225.

[22] Keteyian S. J. , Franklin B. A. , Balady G. J. , Ades P. A. , Bittner V. A. , Beatty A. L. , Beckie T. M. , Brawner C. A. , Brewer L. C. , Brown T. M. , Forman D. E. , Gordon N. F. , Houston Miller N. , Kaminsky L. A. , King M. , Kitzman D. W. , Martin S. S. , Olson T. P. , Pack Q. R. , Pasquali S. K. , Price R. J. , Thomas R. J. , Whooley M. A. , Williams M. A. , and Wu W. C. , ACC/AHA Guideline for Cardiac Rehabilitation, Circulation. (2022) 2022, no. 146, e153–e309, 10.1161/CIR.0000000000001091.

[23] Piepoli M. F. , Hoes A. W. , Agewall S. , Visseren F. L. J. , Mach F. , Smulders Y. M. , Carballo D. , Koskinas K. C. , Bäck M. , Benetos A. , Biffi A. , Boavida J. M. , Capodanno D. , Cosyns B. , Crawford C. , Davos C. H. , Desormais I. , Di Angelantonio E. , Franco O. H. , Halvorsen S. , Hobbs F. D. R. , Hollander M. , Jankowska E. A. , Michal M. , Sgoubopoulou V. , and Stephens J. W. , 2021 ESC Guidelines on Cardiovascular Disease Prevention in Clinical Practice, European Heart Journal. (2021) 42, no. 34, 3227–3337, 10.1093/eurheartj/ehab484. 34458905

[24] Grace S. L. , Turk-Adawi K. I. , Contractor A. , Atrey A. , Campbell N. R. C. , Derman W. , Ghisi G. L. M. , Sarkar B. K. , Yeo T. J. , Lopez-Jimenez F. , Buckley J. , Hu D. , and Sarrafzadegan N. , Canadian Cardiovascular Society Position Statement on Cardiac Rehabilitation, Canadian Journal of Cardiology. (2020) 36, 1847–1864, 10.1016/j.cjca.2020.06.012.33191198

[25] Bauer S. R. , Gellatly R. M. , and Erstad B. L. , Precision Fluid and Vasoactive Drug Therapy for Critically Ill Patients, Pharmacotherapy: The Journal of Human Pharmacology and Drug Therapy. (2023) 43, no. 11, 1182–1193, 10.1002/phar.2763, 36606689. PMC10323046 36606689

[26] Parada-Gereda H. M. , Pardo-Cocuy L. F. , Avendaño J. M. , Molano-Franco D. , and Masclans J. R. , Early Mobilisation in Patients With Shock and Receiving Vasoactive Drugs in the Intensive Care Unit: A Systematic Review and Meta-Analysis of Observational Studies, Medicina Intensiva. (2025) 49, no. 4, 193–204, 10.1016/j.medin.2024.09.002, 39551690. 39551690

[27] Enriquez Popayan A. M. and Gutierrez-Arias R. , Early Active Mobilization in Critically Ill Patients on Vasopressor or Inotropic Support: A Prospective Cohort Study, Journal of Cardiac Critical Care TSS. (2025) 9, 211–218, 10.25259/JCCC_40_2025.

[28] Dexheimer Neto F. L. , Vesz P. S. , Cremonese R. V. , Leães C. G. , Raupp A. C. , Rodrigues Cdos S. , de Andrade J. M. , Townsend Rda S. , Maccari J. G. , and Teixeira C. , Out-of-Bed Extubation: a Feasibility Study, Revista Brasileira de Terapia Intensiva. (2014) 26, no. 3, 263–268, 10.5935/0103-507X.20140037, 25295820. 25295820 PMC4188462

[29] Enríquez Popayán A. M. and García-Agudelo L. , Extubation Outside the Usual Chair Position: Is It Possible? Case Report, Journal of Mechanical Ventilation. (2025) 6, no. 2, 102–106, 10.53097/JMV.10129.

